# Improvement of the Educational Process by Computer-based Visualization of Procedures: Randomized Controlled Trial

**DOI:** 10.2196/jmir.6.2.e16

**Published:** 2004-06-02

**Authors:** Manuel Enzenhofer, Hans-Bernd Bludau, Nadja Komm, Beate Wild, Knut Mueller, Wolfgang Herzog, Achim Hochlehnert

**Affiliations:** ^1^Department of General Internal and Psychosomatic MedicineUniversity of HeidelbergMedical University HospitalHeidelbergGermany

**Keywords:** Computer-based visualization, evaluation of visualization, patient empowerment, technology assessment

## Abstract

**Background:**

Before any invasive procedure, physicians have a legal obligation to inform patients. Traditionally, this involves a discussion with a physician, supplemented by written leaflet information directed at the specific procedure.

**Objective:**

Comparison of the use and effectiveness of computer-based visualization opposed to standardized conversation for providing patients with information of forthcoming procedures (coronary catheters or endoscopy procedures).

**Methods:**

Prospective, randomized trial with 56 participants allocated in two different groups: Visualization Group (standardized information supported by a tool for displaying two-dimensional pictures to explain medical facts as well as informative leaflet) or Control Group (standardized information and informative leaflet only). Detailed information was given about the indication, the probable complications and the details of the forthcoming procedures (coronary catheters or endoscopy procedures). All participants had to reach a Karnofsky Score of 70 points and be able to understand German or English. Main outcome measures were patient's satisfaction with physician-patient conversation, patient's acquired knowledge and duration of the intervention as described above.

**Results:**

Patients of the Visualization Group were more satisfied with the conversation and had higher knowledge scores after the conversation. A Mann-Whitney-U-Test between the two groups showed that these differences in satisfaction (P<0.001) and knowledge (P=<0.006) were statistically significant. Length of time needed for the conversation was slightly higher in the Visualization Group, but this difference was not statistically significant (25 versus 23 min; *P*= 0.441). No differences could be found due to differing age or educational level in the results of the Visualization and the Control Group.

**Conclusions:**

Using computerized visualization increased the satisfaction and knowledge of the patients. The presentation of the visualized information in the Visualization Group did not demand significantly more time than the standard conversation in the Control Group.

## Introduction

### Background

During the last decade we have observed an increasing demand for better integration of patients in clinical and ambulatory health care [1]. Well-informed patients are better able to support their health and to use health services in a sensible way, thus contributing to their treatment outcome. Patients need more possibilities to keep themselves informed about medical benefits and the quality of medical care [2,3].

The information has to present the available evidence in a form that is acceptable and useful [4].

Since the beginnings of human communication, learning and comprehension have always been supported graphically. In education, pictures often clarify difficult facts better than written language. In anatomy, for example, drawings by Netter explain the human body [5].

### Patient Information Systems

Educational materials designed to deliver information and support a more active participation of patients in health care decisions can be effective tools for empowering patients [6]. Shaw et al found that in patients having colonoscopies,computer-assisted instruction (CAI) provided better comprehension and greater satisfaction with computer-assisted education than standard education [7]. Another randomized and controlled trial aimed to determine the impact of an interactive diagnosis-specific video program for informing patients about possible treatments on outcomes and surgical choices. The tested program facilitated decision-making and helped to ensure informed conversation [8]. As a result, standardized templates and systems of informative and visual material are increasingly used to inform patients [4,9]. A distinction has to be made between passive and active (interactive) systems [10,11]. The conventional paper-based patient information brochure is a typical example of a passive system [12]; others are web-based (WWW) information tools, which are gaining more and more importance [13].

#### Patient Information Systems Used by Physicians

**Figure 1:**
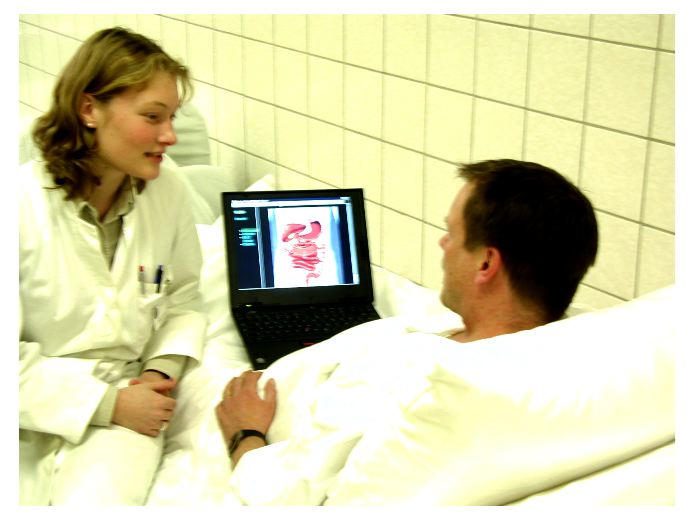
Mobile computer at patient bedside

Most multimedia tools and information brochures serve as a source of information for patients lacking a professional adviser. It has been reported that in too many cases the information contained in patient information leaflets is inaccurate or misleading [4]. The issues of a possible time-pressure of the advisor, the variety of differential diagnoses, and the problems with language barriers and social circumstances raise the question of how the physician is to render comprehensible information to the patient [1,14]. Coulter reported that physicians who are concerned that more empowerment for patients means greater burdens on their time should consider ways of sharing the load. She pointed out that information material and educational packages are available to help in this task [15]. Through using an active system-like our tested system "Dr Topf's patient information system"-the physician can control the tool and only show selected pictures to the patients (Figure 1). This system is designed to be used in cooperation with the patient and is expected to lead to better communication and relationship between doctor and patient.

## Materials and Methods

### "Dr Topf's Patient Information System"

**Figure 2:**
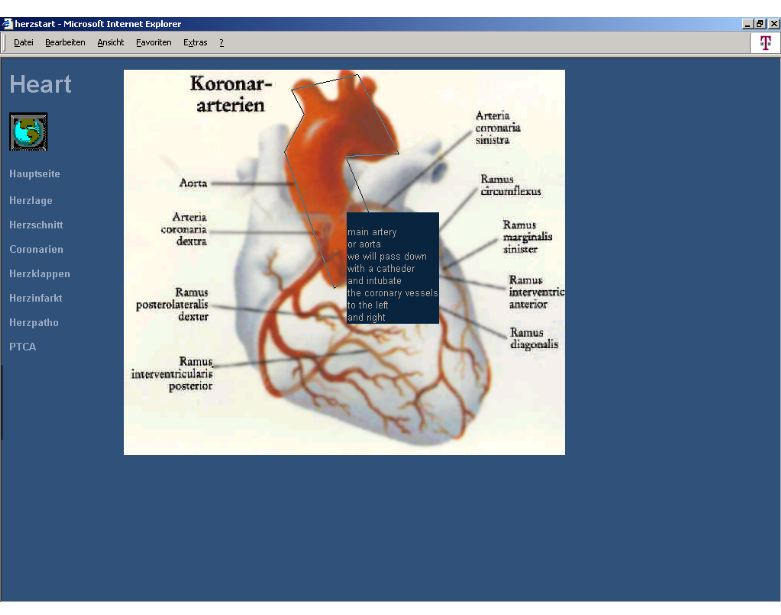
Screenshot Dr Topf

Dr Topf's patient information system makes use of the graphical presentation of medical content during the conversation between the physician and the patient to give the patient a quick and extensive understanding of the medical facts [16]. The system has been developed in cooperation with a scientific institute of general practitioners in Heidelberg in order to explain medical facts with the help of two-dimensional pictures [10]. The browser-based information tool contains a collection of pictures used in cardiology and gastroenterology, and has been primary tested in pilot studies in which 28 patients were informed about their symptoms and the forthcoming procedures [17]. By pointing on different items in these pictures, a short explanation is displayed (Figure 2). The evaluation used standardized questionnaires.

### Protocol

#### Study Population

**Figure 3:**
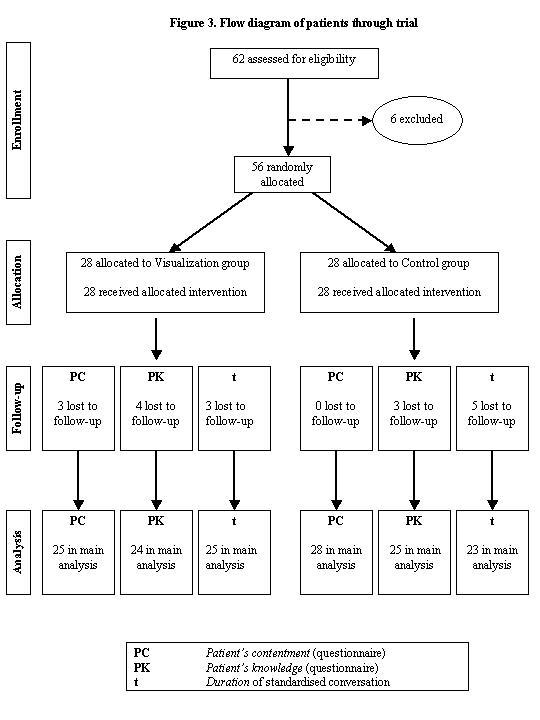
Flow diagram of patients through trial

Participants included 56 patients of a cardiology ward and a gastroenterology ward. The patients were examined over a period of 5 months (Figure 3). Participants needed a Karnofsky Performance Status of 70 points minimum [18] to ensure that they were in the necessary state of health for finishing follow-up. According to this index, they should be able to care for themselves, be neither disabled nor have any serious visual defect, and be literate. Sufficient knowledge of the German or English language was also a criterion of inclusion (55 German, 1 English). In an explanatory document patients were informed that the study would not have any negative effects for them and the law for data protection would be strictly observed.

#### Sample Size

The sample size calculation was determined by the measured effects of our pilot study. As a result of the pilot study, satisfaction and knowledge of the patient obtained effect sizes between 0.65 and 0.71. For a parametric test comparing two independent groups with an assumed power of 0.8 and a level of significance (alpha = 0.05), 26 patients were perceived as an ideal number for each study group [19]. To compensate a loss due to nonevaluable patients, the study was designed to enroll 28 patients per group. The nonparametric test was not adjusted because the collected data did not follow any evident distribution.

#### Assignment and Randomization

For the allocation of the patients to one of the study groups, every physician received eight sealed envelopes. The inscription on the envelopes only indicated the name of the physician and the kind of procedure (cardiology or gastroenterology). Four of the envelopes contained method A (standardized information supported by computer-based picture material), the other four contained method B (standardized conversation). The proportion of four patients per intervention group was equally divided into cardiology and gastroenterology procedures. To prevent the case of double information, all physicians were told that one of their envelopes had been given to another physician. This implied that the ratio of method A to method B could have changed from 4:4 to 5:3 or 3:5. As a consequence the physician would remain blinded from his first to his last patient. In the course of this study no change of the ratio was needed, so a balance of 4:4 for each physician was guaranteed.

#### Intervention

After signing a written declaration of consent, patients were randomly assigned to one of two groups via the random envelopes as previously described . The intervention group received standardized information supported by picture material (i.e. a sample of five pictures maximum was presented on a sub-notebook at patient bedside). The computerized presentation was limited to 5 minutes. Physicians who were taking part in the study had been trained to handle the information tool before the trial began.

A second group was informed by means of standardized conversation by a physician. This group was referred to as the "Control Group" because this procedure is the most common way of informing patient in Germany. Participants of both groups received the same informative brochure [12].

Seven physicians (4 senior house officers, 2 residents, 1 junior house officer) had to inform four patients of each group. Before providing information to the patient, they had to report to the study supervisor whether a patient met the criteria of inclusion.

The physicians gave every participant detailed information about the indication, the probable complications, and the details of the forthcoming procedures (i.e. about anatomy, pathology, complication ratio, possible side effects, postinterventional behavior, and alternative interventions). The following procedures were taken into consideration:

*Cardiology procedures:*

- Right-cardiac catheter
- Left-cardiac catheter and coronary catheter
- Percutaneous transluminal coronary angioplasty (PTCA)
- Electrophysiologic catheter of the right heart

*Endoscopy procedures:*

- Endoscopic retrograde cholangiopancreatography (ERCP)
- Gastroscopy
- Colonoscopy

A list with all necessary contents regarding each procedure was given to the physicians.

Consequently, they had to give every participant detailed information about the purpose of the procedure (pathological changes such as ulcers, varicose veins, sources of bleeding, polyps, or tumors), alternative ways to the procedure (e.g., x-ray, surgery), the probable complications and their treatment (e.g., punctured or injured colon wall requiring immediate surgery; bleeding, which can be treated by injection of drugs; allergic reactions), and the appropriate postsedation behavior (bed rest, no food or liquids for at least 1 hour after the examination).

The procedure was carried out one day after providing the information to the patient.

### Outcomes Measured

After the intervention, every physician completed an anonymous numbered protocol to determine the time spent on the conversation, the time used for visualization, the method of intervention, the kind of procedure, and any important questions asked by the patient.

**Figure 4:**
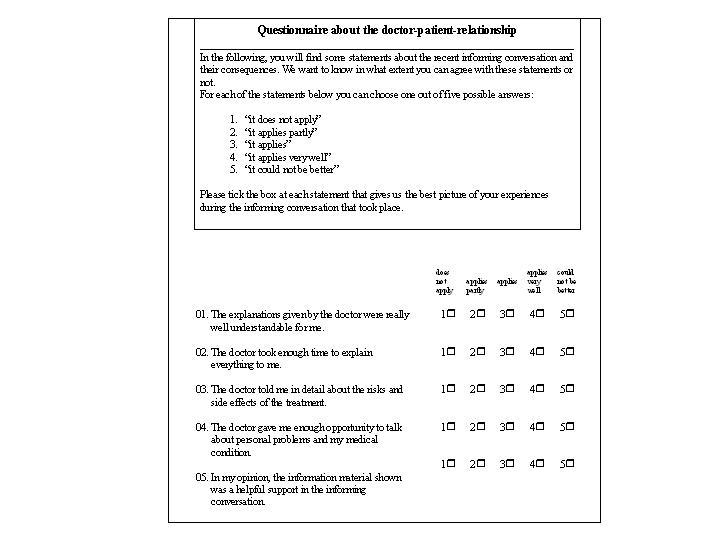
Patient satisfaction questionnaire

Shortly after the conversation the patient was asked to personally assess the quality of the physician-patient conversation via a patient satisfaction questionnaire (Figure 4). Five possible answers ranging from "it does not apply" (one point) to "it could not be better" (five points) were arranged on an ordinal scale. A total score of 5 to 25 points could be reached. Higher scores indicate greater satisfaction (see Table 2).

The evidence of the visualized approach was evaluated using a formalized questionnaire (standard of knowledge). Ten multiple choice questions taken from assessment papers for medical students and adapted to patient knowledge level were used to assess the method of patient education. For every query the patient had to choose either a correct or a wrong statement of five probable statements. A total score of 10 points could be reached. Higher scores indicate greater knowledge (Table 2). The questionnaires had to be answered within three days after the intervention.

### Statistical Analysis

Scale reliability was calculated for patient's satisfaction as internal consistency (Cronbach's alpha coefficient) for the total sample population. Baseline data was collected before randomization. To check whether the assessment criteria correlated with the patient's educational level, age, and the time allocated to the conversation, a Mann-Whitney U test or *t-* test for unrelated groups using the SAS System®, version 8.2 was performed [20].

The Mann-Whitney U test was used for the comparison of patients' satisfaction and knowledge in both groups.

### Masking

Allocations were sealed in opaque numbered envelopes that were opened by the physician after instruction by the independent study supervisor who generated the allocation sequence. Questionnaires had been handed out to the patients by an independent observer who was not informed about which group each patient was in. The statisticians had no contact with study participants and received only unblinded data.

## Results

### Participants Flow and Follow-Up

Between June and October 2002, a total of 62 patients were identified as potential participants. Of the remaining 60 patients who met the criteria of inclusion, 56 received the allocated intervention (see Figure 4). One patient received English information because of minor knowledge of the German language. Three patients dropped out because of moving to other department, 3 other patients did not meet the criteria of inclusion as acute distress (2 patients) and anxiety (1 patient). Eighty-eight percent completed the multiple choice questionnaire and 95% returned the patient satisfaction questionnaire. The reasons given for not completing follow-up (patient's satisfaction and knowledge) were not specific and included inconvenience (2 patients), lack of interest (2 patients), acute depression (2 patients), and moving to another department (4 patients).

The length of time needed for the conversation was analyzed for 86% interventions (see Table 2). No patients exceeded the limited time for visualization (5 minutes maximum).

### Baseline Characteristics

As the performed *t*-test showed, no major differences were seen between the characteristics of the 56 patients of the Visualization Group and the Control Group. Furthermore, no significant difference resulted from professional qualification.

The study subjects ranged in age from 22 to 91 years. The average age was approximately 57.5 (SD 13.8) years (Table 1).

**Table 1:** Sociodemographic data of the Visualization- and Control Group

Variable		Visualization Group *(n=28)*	Control Group *(n=28)*	P*
Age (years)				0.498
	Mean age ± SD†	55.7 ± 10,35	58.2 ± 11.6	
Gender				0.717
	Female	11	8	
	Male	17	20	
Professional qualification				0.666
	N.A.†	3	1	
	Apprenticeship	9	18	
	Craftsman/technical school	6	2	
	Technical college/university	3	3	
	No graduation	7	4	

^*^ The group differences were calculated using *t* test (age), Mann-Whitney-U-Test (professional qualification) and χ^2^ test (gender) at the 5% level of significance.

^†^ SD = standard deviation; N.A. = not announced

**Table 2:** Outcome measures of the Visualization- and Control Group

	Visualization Group *(n=28)*			Control Group *(n=28)*			
Variable	*n*	*M (CI)*	*SD*	*n*	*M (CI)§*	*SD§*	P*
Patient satisfaction questionnaire‡	25	21.2 (19.2 to 23.8)	4,8	28	15.8 (14.1 to 17.5)	4,5	<0.001
Item no. 5		4.1 (3.50 to 4.69)	1.44		2.9 (2.46 to 3.34)	1.13	
Knowledge questionnaire†	24	7.21 (6.5 to 7.9)	1,6	25	5.04 (3.3 to 6.2)	2,8	0.006
Knowledge questionnaire†	24	7.21 (6.5 to 7.9)	1,6	25	5.04 (3.3 to 6.2)	2,8	0.006
Knowledge questionnaire†	24	7.21 (6.5 to 7.9)	1,6	25	5.04 (3.3 to 6.2)	2,8	0.006
Overall time *(min)*	25	10.16 (8.55 to 11.24)	3,0	23	9.23 (7.19 to 11.28)	4,8	0.441
Time for visualization *(min)*		3.54 (3.41 to 4.40)	1.2				

^*^ The group differences were calculated using *t* test (overall time) and Mann-Whitney-U-Test (patient satisfaction - and knowledge questionnaire) at the 5% level of significance.

^‡^ possible range 5 - 25 points

^†^ possible range 0 - 10 points

^§^ M = mean score; CI = 95% confidence interval, SD = standard deviation

### Primary Outcomes

Cronbach's alpha coefficient for the internal consistency of the patient satisfaction questionnaire was 0.94 and can be considered good (Figure 4). An evaluation of the satisfaction questionnaire showed a difference of 5.4 points between the Control and Visualization Groups (95% confidence interval [CI] = [2.9 to 7.9]). Concerning the evaluation of the informative material, emphasis should be given to the fact that patients of the Control Group only awarded 2.9 points compared to 4.1 points awarded by the Visualization Group (95% - CI = [0.95 to 1.45]).

In the total knowledge score, the patients of the Visualization Group reached 2.2 points more than the patients of the Control Group (95%-CI = [0.9 to 3.43]) (see Table 2).

No major differences were seen between the length of time needed for the conversation of the analyzed 48 patients of the Visualization and Control Groups (average time, *P*= 0.441) (see Table 2).

### Qualitative Data

Textbox 1 shows some of the spontaneous comments from physicians.

**Quotes from physicians after the conversation**

"I do not feel that a presentation of the images takes up significantly more of my time. However, as a consequence, the patient wants to learn more about his disease from the physician."

"The laptop computer did not attract the attention of the patient too much. I had the impression that the patient quickly picked up the physiological-pathological information and was able to ask further specific questions."

"Letting the physician operate the program seems more effective to me than having the patient look at such images by himself."

## Discussion

In this prospective, randomized trial, we hypothesized that computer-based visualization would support a conversation for providing patients with information about forthcoming procedures. The patient's satisfaction with the conversation revealed higher satisfaction scores. In spite of the high reliability score of the internal consistency (0.94), sufficient variance in the scale of the patient satisfaction questionnaire was found. As a main focus, the impact of the computer-based visualization tool was directly addressed by our questionnaire, which showed a difference of 1.2 points. This means a difference from "it applies" (3 points) to "it applies very well" (4 points).This observation is consistent with other reported results [7,21].

The time needed for the conversation between physician and patient when supported by visualization was one of the most important points of interest. Some physicians pointed out that the supplement of visualization did not take more time compared to the standardized conversation (see Table 2). Although they needed more time for instruction with the computer-based information, overall time possibly could be reduced because patients could work with the computer-based information mostly by themselves. Additionally, one physician stated that these patients seemed to have less questions than the patients in the Control Group.

While in the present study the software ran on a laptop computer and was brought to the patients' bedside, the information, which is implementated in HTML, could alternatively be distributed to patients via the internet prior to hospitalization. In the future, patients undergoing elective procedures could be empowered at home or in the general practitioner's office before hospitalization. In this study our main focus was on the examination of patient empowerment by physicians assisted with computer-based visualization for already hospitalized patients.

By the increase of knowledge in the Visualization Group, it could be assumed that visualization effectively supports the educational process. Although other studies have evaluated patient satisfaction with computer-assisted instruction, few have evaluated patient knowledge before forthcoming procedures [7]. In this study patients informed by support of visualization scored significantly higher on the knowledge scale than patients from the Control Group (Table 2). The question of whether patients really get a better understanding of the medical content by being informed with support of visualization certainly depends on their previous knowledge and their intellect. A standardized questionnaire about intellect was not performed because of limited time (other examinations of the patient before forthcoming procedure), but there was no significant difference in regard to professional qualification.

Another concern frequently voiced by physicians during the pilot phase of testing was that the visualization could raise patients' anxiety. In this study none of the patients mentioned or expressed concerns in any other way that would support this hypothesis.

After our pilot study we decided to continue the study with several physicians and one independent observer to minimize the Hawthorne-Effect [22]. In contrast to our initial assumption that the computer could be an obstacle for the interaction between patient and physician, we observed the opposite effect: intrigued by visualization, patients asked more questions about the forthcoming procedures (see Box 1). Accordingly, the computer helped to improve the communication between patient and physician and reduce some of the differences in knowledge, especially for patients with little knowledge of medicine. This suggests that active patient information systems such as "Dr Topf´s patient information system" have a significant role in promoting shared decision-making. By assisting patients in clarifying and expressing their values and preferences even when their physicians have different values and preferences, such visualization is a step toward a better relationship between patient and physician.

Our findings show that computer-based visualizations like "Dr Topf´s patient information system" have desirable effects on the patient's satisfaction and knowledge. Research into improving health care by visualization of medical content should be intensified. Following the line of argumentation of Faden and Beauchamp [23] and the principles of the "informed consent" of patients, we showed the feasibility of computer-based visualization within the same time, compared to the paper-based standard, with our patients achieving higher levels of satisfaction and knowledge.
